# The Contribution of Phonological Awareness to Reading Fluency and Its Individual Sub-skills in Readers Aged 9- to 12-years

**DOI:** 10.3389/fpsyg.2017.00533

**Published:** 2017-04-11

**Authors:** Zena Elhassan, Sheila G. Crewther, Edith L. Bavin

**Affiliations:** Department of Psychology and Counselling, La Trobe University, BundooraVIC, Australia

**Keywords:** reading fluency, phonological awareness, FastaReada, phonological decoding, visual word recognition, reading rate, reading comprehension

## Abstract

Research examining phonological awareness (PA) contributions to reading in established readers of different skill levels is limited. The current study examined the contribution of PA to phonological decoding, visual word recognition, reading rate, and reading comprehension in 124 fourth to sixth grade children (aged 9–12 years). On the basis of scores on the FastaReada measure of reading fluency participants were allocated to one of three reading ability categories: dysfluent (*n* = 47), moderate (*n* = 38) and fluent (*n* = 39). For the dysfluent group, PA contributed significantly to all reading measures except rate, but in the moderate group only to phonological decoding. PA did not influence performances on any of the reading measures examined for the fluent reader group. The results support the notion that fluency is characterized by a shift from conscious decoding to rapid and accurate visual recognition of words. Although PA may be influential in reading development, the results of the current study show that it is not sufficient for fluent reading.

## Introduction

Reading fluency is a complex concept that has been defined as “a level of accuracy and rate where decoding is relatively effortless… and where attention can be allocated to comprehension” (Wolf and Katzir-Cohen, 2001, p. 219). Fluency develops with experience derived from biological and cognitive processes related to orthography (visual), phonology (sound), semantics (meaning), and context (background knowledge) (Reichle et al., 2003; Cheng and Caldwell-Harris, 2016; Schuster et al., 2016). However, rather than focusing on fluency, much of the reading literature has reported on the link between reading and phonological awareness (PA), especially among beginning and poor readers. Indeed, a large body of research exists linking PA skills with word reading (e.g., Liberman, 1971; Wolf et al., 2002; Vellutino et al., 2004; Ziegler and Goswami, 2005). PA refers to the ability to recognize individual letters and their correspondence with sounds, and is the basis for decoding spoken words into phonemes (the smallest sound units of language), syllables (segments of speech that are uninterrupted by obstructions to airflow), onsets (the initial sound of a word), and rimes (the unit that follows the onset) (Roach, 2000; Walton and Walton, 2002). PA is a component of the broader category of phonological processing, which refers to the use of speech sounds in processing written language (i.e., reading, spelling) and oral language (i.e., listening, speaking) (Wagner and Torgesen, 1987). Phonological processing also encompasses phonological working memory skills and lexical retrieval.

Several theories of reading acquisition have modeled the development of reading skills on a continuum that consists of multiple phases, each characterized by a specific decoding strategy. Frith’s (1985) influential three-phase model of reading acquisition is characterized by the logographic phase, the alphabetic phase, and the orthographic phase. The logographic phase is associated with pre-readers who have not yet commenced formal reading instruction. Children in this pre-literacy phase do not yet have an understanding of the relationship between phonemes and graphemes. Instead, they rely on a visual recognition strategy to identify high frequency words, words with special significance, and logos as whole units (e.g., the golden arches in the McDonalds logo). The alphabetic phase follows the commencement of formal reading instruction, and is characterized by the application of phoneme/grapheme correspondence rules to phonologically decode words. In the orthographic phase, orthographic representations become established in memory as a result of frequent exposure.

Ehri later modified the terminology of Frith’s (1985) model, and divided the alphabetic phase into two parts to create a more explicit four-phase model of reading development (Ehri, 1995, 1998, 1999, 2002, 2005). Furthermore, Ehri’s model emphasizes the influence of alphabetic processing and extends its involvement as a key component of reading development across all four phases. The pre-alphabetic phase has a similar concept to Frith’s logographic phase; however, the term *logographic* was replaced as Frith argued that it is suggestive of a mature reading strategy. The partial alphabetic phase commences when learner readers begin committing phoneme–grapheme associations to memory, and applying this knowledge in attempts at word pronunciation. Children transition to the full alphabetic phase when their alphabetic skills become established, allowing them to accurately decode novel words using a phonological decoding strategy. By this point, decoding experience has contributed to the expansion of ‘sight word’ knowledge, a term used by Ehri to describe the bonding between spelling patterns of whole words and their pronunciations, which allows for visual recognition of words. This account is consistent with the self-teaching hypothesis, which was proposed by Firth (1972), and expanded by Jorm and Share (1983). With reference to the self-teaching hypothesis, Share (2008b) argued that phonological decoding allows learner readers to autonomously build detailed orthographic representations, that can be activated in order to rapidly visually identify words. The final phase of Ehri’s model is the consolidated alphabetic phase, characterized by automaticity, which she defines as, “… recognizing the pronunciations and meanings of words immediately upon seeing them without expending any attention or effort decoding the words” (Ehri, 2005, p. 151). Indeed, with ongoing exposure to text and reading experience, the orthographic lexicon (i.e., long-term memory for orthographic information) continues to expand (Ehri, 2014; Perfetti and Stafura, 2014). The expanding lexicon allows readers to identify words visually within milliseconds; improving fluency and supporting comprehension (Levy et al., 1997; Rayner et al., 2001). Thus, reading fluency is indicative of a shift from phonological decoding to rapid, visual word recognition (Dietz et al., 2005). Certainly, it has been shown that reading speed is limited by increasing reliance on phonological decoding (Elhassan et al., 2015).

The influence of PA to reading skills in fluent readers has had limited attention in the literature, with many studies investigating PA in reading disordered groups in comparison to typically achieving groups (Casalis et al., 2004; Katzir et al., 2006; Constantinidou and Stainthorp, 2009). The current study was designed to address this gap. The aims of the study were to demonstrate that reading fluency is a fast automatic visual process, and to investigate the extent to which PA continues to predict reading outcomes in fluent readers.

Children’s PA skills are often measured with several standardized measures. For example, the Comprehensive Test of Phonological Processing (CTOPP) uses three tasks to provide an overall indication of PA skills through the PA composite measure (Wagner et al., 1999). Sound Matching assesses the ability to identify words with corresponding sounds at the beginning or ending sounds (in children aged 5–6 years); Elision examines the ability to omit (i.e., elide) a phonological unit from spoken words to produce another word; Sound Blending assesses the ability to combine sounds to form words. According to Stanovich’s (1992) conceptualization, children initially acquire a shallow sensitivity for large phonological units, such as words and syllables. Over time, phonological sensitivity progresses to onsets and rhymes, eventually reaching a “deep” awareness of small phonological units that allows for the manipulation of phonemes. In line with this theory, Anthony et al. (2003) found that blending skills develop prior to elision skills for phonological information of equivalent linguistic difficulty in 2- to 8-year-old children. Previously, however, elision was identified as a stronger predictor of reading skills than Blending (Wagner et al., 1999).

The current study examined the extent to which scores from a combined measure of elision and sound blending for words contributed to phonological decoding, visual word recognition, reading rate, and reading comprehension in a sample of 9- to 12-year-old children grouped on the basis of reading fluency level. Children of this age (fourth to sixth grade) were sampled because according to Jenkins et al. (2003), typically developing readers begin to exhibit larger individual differences in fluency from fourth grade. The research questions were:

(1) Does elision correlate better with reading fluency than sound blending?
(2) Do fluent readers have better developed PA skills than dysfluent readers?
(3) Does the contribution of PA skills decrease with increased reading fluency?

## Materials and Methods

### Participants

The study was approved by the La Trobe University Human Ethics Committee (FSTE HEC 13/R22). Consent to collect data from schools was also provided by the Victorian State Department (2012/001425) and Catholic Education Melbourne (GE12/0009 1765). Permission to test in the schools was given by the school principals. The current study utilized a non-probability sampling method. Mainstream schools in the North East Metropolitan region of Melbourne, Australia were informed about the current study via email and invited to participate. Four schools expressed interest and allowed the author to recruit their students. One hundred and twenty-nine children between the ages of 9- to 12-years were recruited from three grade levels, Grade 4 to Grade 6. In Melbourne, formal schooling begins with ‘Prep’ (preparatory year) then proceeds with primary (Grade 1 through to Grade 6). The schools represented high and low socioeconomic locations. Informed consent was obtained from the child’s parent or legal guardian prior to participation in the study. Participant information and consent forms were disseminated to parents and legal guardians of all children in the target year levels. Every child who returned a signed consent form was permitted to sit the entire battery of assessments to avoid leaving any child with a sense of exclusion. However, for inclusion in data analysis participants required a score above the 10th percentile on the Raven’s Colored Progressive Matrices (RCPM: a test of non-verbal reasoning ability; Raven et al., 1998), adequate or adequately corrected vision and hearing, and typical sensory, mental, and motor development. Three children were excluded from analysis for scoring at or below the 10th percentile on the RCPM. A further two were excluded on the basis of teacher report of confirmed or suspected neurodevelopmental disorder other than DD (Asperger syndrome). The final number of participants was 124 (see **Table 1** for demographics).

**Table 1:** Descriptive statistics between dysfluent, moderate and fluent readers.

	*N*	Age (years)			Grade Level			Sex		RCPM			
					
		Minimum	Maximum	Mean	4	5	6	Male	Female	Minimum	Maximum	Mean	*SD*
Dysfluent	47	9.42	12.83	10.75	18	18	11	18	29	25	35	30.68	2.87
Moderate	38	9.08	12.50	10.83	15	10	13	19	19	26	36	31.97	2.69
Fluent	39	9.58	12.50	11.25	9	13	17	18	21	29	36	33.26	2.01
All readers	124	9.08	12.83	10.92	42	41	41	55	69	25	36	31.89	2.77


*RCPM, Raven’s Colored Progressive Matrices (measure of non-verbal reasoning).*

### Materials

#### Phonological Awareness

##### Phonological Awareness Composite: Comprehensive Test of Phonological Processing (CTOPP)

Phonological awareness skills were assessed with the PA composite of the CTOPP. The children completed the two age-appropriate subtests from the CTOPP required to obtain a composite score, one of which assessed elision, and one of which assessed blending. Both subtests were administered according to standardized instructions in the CTOPP examiner’s manual (Wagner et al., 1999). For the elision subtest the investigator asked the child to say a word. After repeating the word, the examiner asked the child to say the word again without a specified sound (e.g., “Say *toothbrush* without saying /tooth/”, “Say *tiger* without saying /g/”). For the blending words subtest, the investigator asked the child to listen to a word presented in small sound parts, and then to blend the sounds together to form the whole word (e.g., “What word do these sounds make? /b/, /ag/).

The CTOPP provides reliable and valid measures of PA skills as shown by moderate to high internal consistency for Elision (0.81–0.92) and Sound Blending (0.78–0.89) across age groups. The PA composite of the CTOPP also shows high reliability high for both time sampling and inter-scorer differences, strong correlations with the Test of Word Reading (i.e., criterion related validity), positive correlations between age and score (construct validity), and an adequate correlation between test items (content validity) (Wagner et al., 1999).

#### Reading Skills

##### FastaReada

FastaReada is a computer-generated measure of reading fluency (Elhassan et al., 2015). The program presented six-words per trial from an extract of a contemporary children’s novel, which appeals to children aged between 9- to 12-years of age. Children were asked to report the words presented aloud. The investigator pressed one of two keyboard buttons to indicate accurate or inaccurate responses at the end of each trial. The presentation time for each group of words was controlled via the parameter estimation by sequential testing (PEST) adaptive staircase algorithm based on a maximum-likelihood threshold estimation. Children informed that stimuli exposure might become so quick that they would have time to read all six words; however, they were encouraged to attempt each trial even if this was to occur (i.e., recall the trial from memory). This feature is a particular strength of FastaReada compared to typical words read correctly per minute measures as it reduces the impact of motor limitations associated with verbalizations on responding. FastaReada therefore allows for more accurate representations of individual differences in reading ability.

Criterion related validity has been demonstrated with strong positive correlations between FastaReada and the established Neale Analysis of Reading Ability – Third Edition (NARA-3) subtests of reading rate (*r* = 0.75), accuracy (*r* = 0.83), and comprehension (*r* = 0.63). Further evidence of criterion related validity has been shown in strong positive associations between FastaReada performance and performance on the established Dyslexia Determination Test (DDT) measures of eidetic (*r* = 0.81) and phonetic (*r* = 0.68) decoding skills (Elhassan et al., 2015).

##### Pseudoword Decoding: Wechsler Individual Achievement Test–Second Edition (WIAT-II)

The Pseudoword Decoding subtest of the WIAT-II was used to assess phonological decoding. It consists of 54 pronounceable non-word items. The task was administered in line with the general assessment procedure outlined in the WIAT-II manual (Wechsler, 2007). In summary, the investigator asked the children to read each item on the stimulus card, from left to right. All children began at the same starting point and the task was discontinued when seven consecutive incorrect responses were made.

The Pseudoword Decoding subtest has been shown to be a reliable measure of pronounceable non-word decoding skills, with high-level inter-item reliability (0.89–0.98) and test–retest stability (0.93) across ages 6–19 years. Evidence of construct- and criterion-related validity has also been demonstrated across the subtests of the WIAT-II (Wechsler, 2007).

##### Decoding Patterns: The Dyslexia Determination Test (DDT)

The Decoding Patterns subtest of the DDT was utilized to provide a measure of visual word recognition. The task was administered according to the standard procedure for testing (Griffin and Walton, 2003). Children were asked to commence orally decoding the list words from the initial list (i.e., the pre-primer words) rather than the suggested two to three levels below their year level in order to avoid frustration and to assist with building confidence. The items on the list alternate between phonetically irregular words (i.e., requiring visual recognition for accurate decoding) and phonetically regular words (i.e., conforming to English letter-sound rules). In line with the standard DDT procedure, words read correctly within 2 s were marked as eidetic (i.e., visually recognized) as the rapid response is indicative of visual recognition. Words read correctly after a delay of more than 2 s but within 10 s were considered to have required the use of phonics, syllabication and/or structural analysis in word decoding. Words that were not read within 10 s, read incorrectly or not attempted were marked as unknown.

##### Reading Rate and Comprehension: The Neale Analysis of Reading Ability – Third Edition (NARA-3)

The NARA-3 was used to provide an objective measure of reading rate and reading comprehension. It uses Australian norms for children aged 6- to 12-years and provides measures of reading accuracy, comprehension and reading rate (Neale, 1999). The NARA-3 was administered according to the standard procedure for testing, as outlined in the manual (Neale, 1999). In summary, children were instructed to read a series of prose passages presented in book form and answer questions about each passage at its conclusion. Each passage was accompanied by a line drawing that was intended to set the scene rather than to provide detail. The investigator corrected and recorded errors that the children made whilst reading. The investigator also recorded the time taken to read each passage and marked the answers as correct or incorrect. There were six passages in total, which were presented in order of increasing difficulty. Testing was discontinued after the ceiling for reading errors in a passage was reached (16 errors for passages 1–5 or 20 errors for passage 6). Separate scores for accuracy, comprehension and reading rate were obtained.

The NARA-3 has been shown to be a reliable and valid tool for the assessment of reading comprehension. High levels of internal consistency have been demonstrated for the rate (0.73–0.96) and comprehension (0.71–0.96) measures (Neale, 1999) and it has high content and face validity for the construct of oral reading (Neale, 1999). Additionally, it has been shown to have criterion-related validity through its significant correlations with other tests of reading skills (e.g., Moorehouse and Yule, 1974) and through its efficacy at predicting future reading ability (McKay, unpublished as cited in Neale, 1999). Finally, the positive correlation between score and years of schooling provides evidence of construct related validity (Neale, 1999).

#### General Intelligence

##### Raven’s Colored Progressive Matrices (RCPM) Test

The RCPM was used to provide a standardized, untimed, non-verbal measure of general intelligence through the assessment of non-verbal reasoning ability (Raven et al., 1998). The RCPM has Australian norms for typically developing children aged 5–11 years (Cotton et al., 2005). The RCPM consists of three sets of 12 colored multiple-choice items that gradually increase in complexity. Each item consists of an incomplete matrix. Children were asked to identify one of six figures positioned below the rectangle that would correctly complete the pattern. A score of one point was rewarded for each correct answer. The scores were tallied upon completion of the task to provide an overall raw score. Raw scores were converted to percentile scores to rank non-verbal intelligence on the basis of chronological age.

The RCPM has been shown to have good test-retest reliability at *r* = 0.80 (Raven et al., 1998) and high internal consistency (*r* = 0.89), with little variation across age levels (Cotton et al., 2005).

#### Procedure

Data collection took place over three sessions, which totaled approximately 1 h and 30 min for each child. Tests were administered across the three sessions to help maintain interest and to reduce disruptions to classroom learning. They were held during school hours, and within school buildings, typically over three successive school days. The tests were administered in an order that promoted interest and reduced fatigue (i.e., cognitively demanding and paper-based tests were limited in each session and computer-based tests were administered toward the end of each session to act as an incentive). Children were praised for their performances at the conclusion of individual tests and were encouraged to choose a ‘thank you gift’ from a box of novelty stationary items at the end of each session.

#### Statistical Procedures

The Statistical Package for Social Scientists (IBM SPSS Statistics 23) program was used for data screening, data transformations and analysis.

##### Data for the total sample

Outliers and assumption failures were corrected in line with Tabachnick and Fidell (2013). Outliers identified for the reading fluency variable were rescored to the next lowest score identified to reduce influence on the remaining data. The reading fluency variable remained outside normal limits due to positive skewness. A square root transformation resulted in substantial improvement. The negatively skewed distributions of the elision, sound blending and PA composite variables were reflected before transformations were applied (i.e., values were subtracted from a constant calculated by adding 1 to the highest value of the variable). Elision required a log transformation, whilst square root transformations resulted in substantial improvements for the sound blending and PA composite variables. The reflected and transformed variables were then re-reflected to restore the original order of the variables for interpretation, as recommended by Tabachnick and Fidell (2013).

##### Data for reading fluency groups

The reading fluency groups were identified using scores from FastaReada. The FastaReada variable was divided into three equal percentiles based on increasing case values, giving three categories: dysfluent readers, moderate readers, and fluent readers. The PA composite and phonological decoding variables were noted to fall outside normal limits due to negative skewness in the moderate readers group. The phonological decoding variable was also negatively skewed in the fluent readers group. Reflected square root transformation resulted in the normalization of the PA composite variable, whilst a log transformation substantially improved the distribution of the phonological decoding variable. The data was then re-reflected for interpretation.

## Results

### Relationship between Reading Fluency and Different PA Skills

Bivariate Pearson product moment correlations were conducted on the reading fluency, elision, sound blending, and PA composite variables (see **Table 2** for the correlation matrix). A medium^1^, positive association was found between reading fluency and the PA composite (*r* = 0.37, *p* < 0.001), indicating that higher reading fluency scores were associated with higher PA composite scores. A medium, positive association was also found between reading fluency and elision (*r* = 0.39, *p* < 0.001); higher reading fluency scores were associated with higher elision scores. A smaller, positive correlation was also found between reading fluency and sound blending skills (*r* = 0.28, *p* < 0.001); that is, higher reading fluency scores were associated with higher sound blending skills. The means and standard deviations for untransformed elision, sound blending and PA composite variables for each of the reading fluency groups and the total sample are presented in **Table 3**.

**Table 2:** Correlations between FastaReada Scores and elision, blending, and Phonological Awareness Composite Scores for all readers.

	1	2	3	4
(1) Reading fluency	-	-	-	-
(2) Elision	0.38^∗∗^	-	-	-
(3) Blending	0.28^∗∗^	0.43^∗∗^	-	-
(4) PA composite	0.37^∗∗^	0.83^∗∗^	0.77^∗∗^	-


**N* = 124. PA, Phonological awareness. ^∗∗^*p* < 0.01.*

**Table 3:** Means and standard deviations of the CTOPP Phonological Awareness Composite Score and its individual subtests, elision and sound blending by reading fluency group.

Predictor variable	Dysfluent	Moderate	Fluent	All Readers
				
	Mean (*SD*)	Mean (*SD*)	Mean (*SD*)	Mean (*SD*)
Elision	14.43 (4.54)	15.84 (4.82)	17.62 (3.45)	15.86 (4.49)
Sound blending	15.57 (3.70)	16.32 (3.22)	17.90 (1.94)	16.53 (3.22)
Phonological awareness composite	29.79 (7.14)	32.18 (6.81)	34.87 (4.44)	32.12 (6.60)


**N* = 124. CTOPP, Comprehensive Test of Phonological Processing. Untransformed data presented.*

### Relationship between PA Skills and Increasing Reading Fluency Level

To determine whether there were any statistically significant differences between the PA skills for the three reading fluency groups, a one-way ANOVA was used. Assumptions for one-way ANOVA analysis were met and the data were found to be free from outliers, were normally distributed, and had homogeneity of variance. Data are presented as mean ± standard deviation. Effect sizes are reported using partial eta squared ($\eta_{p}^{2}$) for the main effect^2^, and Cohen’s *d* for the simple effects^3^. A Tukey honest significant difference *post hoc* comparison was performed to investigate the differences between the groups.

**FIGURE 1:**
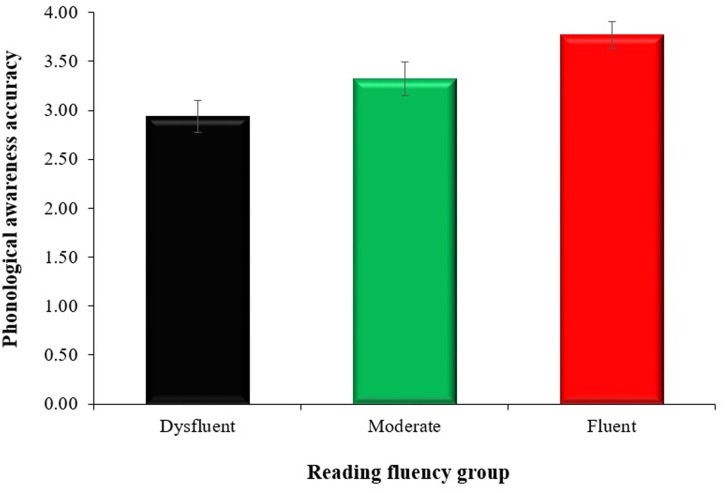
**Mean phonological awareness scores across dysfluent, moderate, and fluent readers.** Error bars represent standard error.

The one-way ANOVA revealed a statistically significant difference among reading fluency groups on the PA composite of the CTOPP with a medium effect size, *F*(2,121) = 7.18, *p* = 0.001, $\eta_{p}^{2}$ = 0.106; PA scores were higher with increasing fluency. Specifically, dysfluent readers had the lowest mean PA score (2.94 ± 1.11), followed by moderate readers (3.32 ± 1.06), and fluent readers (3.77 ± 0.86). Tukey *post hoc* analysis indicated that mean PA scores were significantly higher in the fluent readers than the dysfluent readers (0.84, 95% CI [0.32 to 1.36], *p <* 0.001, *d* = 0.84). The effect size was large for the comparison between dysfluent and fluent readers. Moderate readers did not differ significantly from dysfluent (0.38, 95% CI [-0.14 to 0.91], *p* = 0.201, *d* = 0.47) or fluent readers (0.45, 95% CI [-1.01 to 0.10], *p* = 0.128, *d* = 0.34) in their PA skills, with both sets of pairwise comparisons demonstrating a small effect size. The mean PA scores for the three reading fluency groups are shown in **Figure 1**.

### Contribution of PA Skills to Reading Skills between Reading Fluency Levels

**FIGURE 2:**
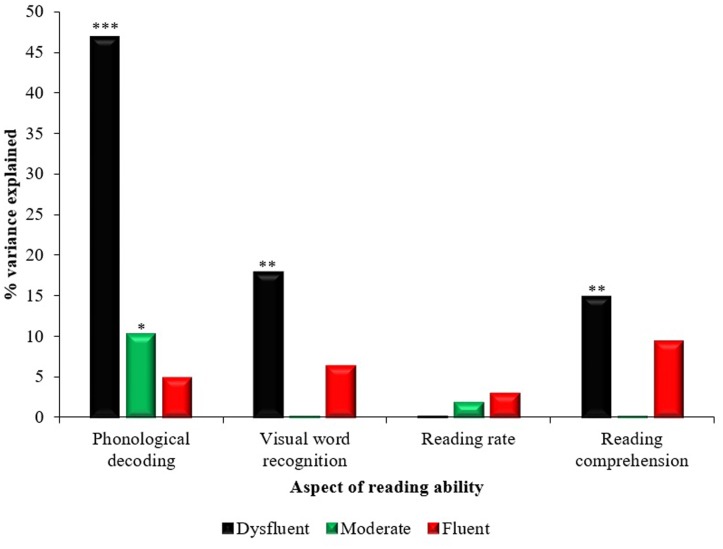
**The contribution of phonological awareness to accuracy of phonological decoding, visual word recognition, reading rate, and reading comprehension across dysfluent, moderate, and fluent readers.** ^∗^*p* < 0.05, ^∗∗^*p* < 0.005, ^∗∗∗^*p* < 0.001.

Simple linear regressions were used to explore the proportion of variance in the dependent variables that could be accounted for by the independent predictor variable (i.e., how well PA predicted performance on measures of phonological decoding, visual word recognition, reading rate, and reading comprehension) for the three reading fluency groups. Twelve simple regressions were run in total, one for each dependent variable in each reading fluency group. Multiple correlation coefficient squared (*R*^2^) values were reported on a range from 0 to 100% to indicate the proportion of variance in reading skills that was accounted for by PA skills. See **Figure 2** and **Table 4** for a summary of the results.

**Table 4:** Results of linear regression analyses examining the contribution of Phonological Awareness to different aspects of reading ability for dysfluent, moderate, and fluent reading groups.

Group	Criterion variable	*t*	β	*F*	*df*	*p*	Adj. *R^2^*
**Dysfluent readers**							
	Phonological decoding	6.29	0.68	39.58	1,45	0.000	0.456
	Visual word recognition	3.13	0.42	9.82	1,45	0.003	0.161
	Reading rate	-0.18	-0.03	0.03	1,45	0.857	-0.021
	Reading comprehension	2.80	0.39	7.85	1,45	0.007	0.130
**Moderate readers**							
	Phonological decoding	2.03	0.32	4.11	1,36	0.050	0.078
	Visual word recognition	-0.26	-0.04	0.07	1,36	0.798	-0.026
	Reading rate	0.81	0.13	0.66	1,36	0.421	-0.009
	Reading comprehension	-0.14	-0.02	0.02	1,36	0.886	-0.027
**Fluent readers**							
	Phonological decoding	1.37	0.22	1.88	1,37	0.179	0.023
	Visual word recognition	1.57	0.25	2.47	1,37	0.125	0.037
	Reading rate	1.08	0.17	1.16	1,37	0.289	0.004
	Reading comprehension	1.95	0.31	3.81	1,37	0.058	0.069


**N* = 124. The independent variable for all regressions was phonological awareness.*

In dysfluent readers PA skills predicted significant variance in phonological decoding (46.8%), visual word recognition (17.9%), and reading comprehension (14.9%) but not reading rate. In moderate readers PA explained 10.2% of the variance in phonological decoding but it did not contribute significantly to the three other reading tasks. For fluent readers, PA skills did not contribute significant variance to any of the four reading measures.

**FIGURE 3:**
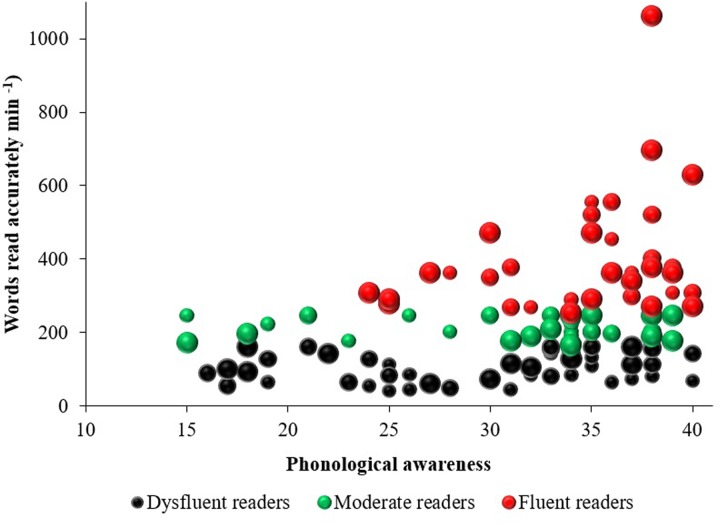
**Words read accurately per minute as a function of phonological awareness between reading fluency groups.** Reading fluency group membership is indicated by dot color. Grade level from Grade 4 to Grade 6 is indicated by increasing dot size. This chart presents untransformed data.

In order to further examine differences between PA skills and reading fluency the relationship between PA and reading fluency scores was plotted for each child. The bubble chart presented in **Figure 3** shows the relationship between PA skills and reading fluency.

As shown in **Figure 3**, there was a positive association between PA scores and reading fluency. However, two of the five readers who reached ceiling on the PA assessment were dysfluent (one from Grade 4, and one from Grade 5). Conversely, the two poorest performers on the PA assessment, who scored only 15 out of 40, were moderate readers (one from Grade 4, and one from Grade 6). Whilst the majority of fluent readers scored in the top third of the PA results range, there were three Grade 6 fluent readers who scored in the middle third of this range, obtaining scores of 24 and 25 out of 40.

## Discussion

The study investigated the influence of PA skills on different aspects of reading in children from Grades 4, 5, and 6 with different levels of reading fluency (dysfluent, moderate, and fluent). A predominant view in the literature has been that reading difficulties are primarily associated with deficits in phonological analysis (Vellutino et al., 2007). However, while PA is an established important predictor of early reading development, it is unclear if it continues to predict reading outcomes in fluent readers. The general finding is that once a child is able to read fluently, PA skills do not, overall, contribute significantly to measures of reading.

Elision was found to be more strongly correlated with reading fluency than blending, a result that complements the earlier findings of Wagner et al. (1999). In keeping with Stanovich (1992), the stronger association between elision and fluency than sound blending and fluency indicates that the development of skilled reading is associated with a “deeper” awareness of phonological units, which, in turn, allows for more efficient segmentation, deletion, and blending of phonemes. This finding implies that elision is a more sophisticated skill than sound blending (Stahl and Murray, 1994; Lombardino et al., 1997; Schatschneider et al., 1999; Kroese et al., 2000), and explains why elision skills develop later than sound blending skills (Anthony et al., 2003). Higher scores on the elision measure are therefore indicative of better developed phonological processing and conceptualization skills.

When the composite PA score was examined between reading fluency groups, fluent readers were shown to have better developed PA skills than dysfluent readers. The implication of this finding is that the fluent readers examined were hypothetically better able to decode regular words than the dysfluent readers. Whilst reading fluency is characterized by fast automatic visual recognition of connected text, this finding supports the notion that acquisition of reading fluency requires automatization of strategies that allow independent decoding of novel words. According to the self-teaching hypothesis, phonological decoding skills represent such a strategy (Firth, 1972; Jorm and Share, 1983; Share, 1995, 2008a). However, whilst PA may play an important role in expanding readers’ orthographic representations of regular words, the relatively high prevalence of irregular words in the English orthography (making it a ‘deep’ orthography) indicates that PA alone cannot be sufficient for the development of fluency.

The contribution of PA to different aspects of reading ability was shown to decrease with increasing fluency. PA played a contributory role in all aspects of reading ability examined in dysfluent readers, except for reading rate. Phonological decoding scores were most influenced by PA skills, followed by visual word recognition, and reading comprehension. This finding implies that dysfluent readers rely more heavily on a decoding strategy for real words that involves segmenting and blending phonemes than moderate and fluent readers. Over-reliance on such a strategy would be expected to lead to errors when reading irregular words. It would also influence the rate of reading as conscious phonological decoding of words is a slower reading strategy than visual word recognition. The influence of PA on the dysfluent group’s reading skills therefore reflects a period between Ehri’s (1995) partial alphabetic phase and full alphabetic phase.

Moderate readers’ visual word recognition, reading rate, and reading comprehension skills were independent of PA skills. In comparison to the dysfluent sample, the influence of PA skills on phonological decoding in the moderate readers group was small. These findings imply that moderate readers fall between the full alphabetic phase and the consolidated alphabetic phase of Ehri’s (1995) model as PA no longer contributes significantly to word identification. However, reading skills are not yet substantially automatized, as demonstrated by the continued influence of PA to phonological decoding. In contrast, PA did not impact on any of the reading skills examined in the fluent readers group, indicating that fluent readers have attained a level of automaticity that significantly reduces their reliance on a conscious phonological-based approach to decoding. The fluent readers’ profile therefore reflects the consolidated phase of Ehri’s model.

The difference found in phonological decoding skills between moderate and fluent readers indicates an important factor in the establishment of reading fluency. Pronunciations of novel or pronounceable non-words can then be generated by analogical reasoning rather than phonological decoding, although the use of analogy could also lead to an incorrect solution (Glushko, 1979; Walton and Walton, 2002; Goswami, 2003). For example, when decoding the irregular word *pint* for the first time, the reader may draw upon the orthographic similarities between *pint* and *mint*, and therefore inaccurately decode it as [pInt] to rhyme with *mint*. By way of pseudowords, the reader may have more success implementing an analogical reasoning strategy when decoding *pragment*, which is pronounced [′prægmǝnt], due to its orthographic similarity to the regular word *fragment*.

The results for the fluent readers group lend support to the findings of de Jong and van der Leij (1999) who conducted a longitudinal study of the contribution of PA to the prediction of reading fluency for words and non-words in a sample of Dutch children. Unlike the findings of Vaessen and Blomert (2010), who noted the influence of PA to extend throughout primary schooling, de Jong and van der Leij (1999) found that PA no longer contributed to word and pseudoword reading speed beyond the conclusion of Grade 1. Vaessen and Blomert’s (2010) results may have been influenced by the use of speeded PA measures. The findings from de Jong and van der Leij (1999) are also in line with those of Aro and Wimmer’s (2003) who showed that learner readers of Dutch, Finnish, French, German, or Spanish orthographies (all transparent, in contrast to English) achieve accuracy levels of approximately 85% in non-word decoding by the conclusion of first grade. Accuracy in Swedish learner readers was noted to reach above 90%. In comparison, learner readers of English obtained accuracy levels of only 50% by the end of first grade; they did not match the accuracy levels of their European peers until fourth grade. Thus, it is likely that once automaticity in reading is attained, there is independence from PA skills when reading new words.

Interestingly, several dysfluent readers had exceptionally well developed PA skills, with two dysfluent readers achieving maximum scores on the PA measure used. Furthermore, several fluent readers only achieved moderate PA scores. These findings support previous reports that not all children with reading difficulties show the predicted phonological deficit (Boder, 1973; Castles and Coltheart, 1993; Wolf et al., 2002; Whiteley et al., 2007; De Luca et al., 2010). Similarly, Snowling et al. (2003) demonstrated that some children with familial risk for Developmental Dyslexia (DD; a neurodevelopmental “specific” reading disorder) do not develop the disorder despite having PA deficits. Such findings indicate that alternative strategies, including visual word recognition, can be used to compensate for PA deficits. Furthermore, although phonological remediation can result in improved single word reading and comprehension skills in individuals with DD, speed and prosody (expression) in oral reading remain elusive (e.g., Ferrer et al., 2010).

One limitation of the study was the small number of participants in each reading fluency group (between 38 and 47 participants). This may have reduced the statistical power of the regression models used to examine the influence of PA in reading abilities between children of different reading fluency levels. Although the unique contributions of PA to reading skills did not approach significance in the regressions that fell outside of significance (with the exception of unique PA contribution to reading comprehension in fluent readers, which was at *p* = 0.058), the question of reduced power for non-significant findings cannot be disregarded. The recruitment of larger samples for such analyses in future studies would provide further credibility to the current findings. Nevertheless, taken together, the current findings demonstrate the importance of considering the characteristics of poor readers in the design of remediation programs. A causal role of PA in the acquisition of reading has been well discussed in the literature and, accordingly, remediation programs have been designed with a strong focus on phonological skill training. However, whilst PA leads to increased decoding accuracy, accurate pronunciation of irregular words may continue to be problematic, and the fast automatic visual method of reading that is associated with fluency is unlikely to develop. An important direction for future studies is consideration of other known contributors, including vocabulary level, to reading skills in different reading fluency groups. This was not examined in the current study; however, vocabulary size has previously been shown to be related to phonological processing skills (Walley et al., 2003; Edwards et al., 2004; Munson et al., 2005).

## Conclusion

The current findings make an important contribution to the literature. By examining the relationship between PA and reading skills for different reading fluency groups, it was found that although PA may be influential in the development of reading skills, it alone is not sufficient for an individual to become a skilled reader. The current study also identified a possible differentiating factor between moderate and fluent readers – the ability to read novel words without reliance on a conscious decoding strategy. Future studies that focus on those skills associated with the fast automatic visual nature of fluency will help strengthen understanding of the risk factors for dysfluent or disordered reading, and thus aid in the implementation of effective educational policies and the development of practical remediation programs.

## Ethics Statement

This study was carried out in accordance with the recommendations of the Australian Code for the Responsible Conduct of Research, the National Health and Medical Research Council with written informed consent from all participants. All participants gave written informed consent in accordance with the Declaration of Helsinki. The protocol was approved by the La Trobe Human Ethics Committee.

## Author Contributions

ZE conceived the study including methodological design, also participant recruitment, data collection, data analysis, interpretation of data, and wrote the manuscript. SC conceived the study including methodological design, also contributed to interpretation of data and critically appraised the manuscript. EB interpreted data, critically appraised and edited the manuscript. All authors approve of this version to be published. All authors take responsibility for the contents of this article.

## Conflict of Interest Statement

The authors declare that the research was conducted in the absence of any commercial or financial relationships that could be construed as a potential conflict of interest.

## Footnotes

1 Effect sizes for bivariate Pearson product moment correlations were interpreted according to Cohen’s (1988, pp. 79–81) conventions. A correlation coefficient of 0.10 is considered a small association, 0.30 is considered a medium association, and 0.50 and above is considered a large effect size.

2 For $\eta_{p}^{2}$, 0.01 is considered a small effect size, 0.06 a medium effect size, and 0.14 a large effect size (Cohen, 1988, pp. 284–287).

3 For Cohen’s *d* 0.20 is regarded a small effect size, 0.50 medium, and 0.80 a large effect size (Cohen, 1988, pp. 25–27).
